# Dental Therapeutics

**Published:** 1876-10

**Authors:** W. E. Driscoll


### DENTAL THERAPEUTICS.

BY W. E. DRISCOLL.

*Read before the Indiana State Dental Association, June 28th, 1876.*

It is hardly expected or desired that I recite to you theories and principles of dental therapeutics generally accepted as correct, and already on record in our professional libraries, and consequently accessible to all who desire to study and improve. I can serve you to better advantage by limiting my remarks to modes of practice that are still questioned by some considerable portion of the profession. I shall further limit myself to those debatable modes that I have thoroughly tested in my own practice, leaving you to decide for yourselves on which side of each question is to be found a preponderance of good authority. In fact, my highest hope in the preparation of this paper will be attained if it leads to a general examination of the subjects touched upon, either here or after your return home.

At our meeting in 1869, amid many expressions of doubt, the claim was made by some of our members, that a considerable proportion of dental pulps could be permanently preserved alive with the new capping os-artificial.

The year following I made quite a number of efforts to save exposed pulps with this material as a capping, but failed, I think, in every attempt.

At the meeting in 1870 the claim of successful experience with this capping was repeated. Upon returning home I adopted the plan of sealing in an exposed pulp that I am about to describe, I had success from the first case. My memoranda of cases is not as full and specific as I wish it was, yet I have enough recorded to state positively that five per cent. will include every failure from every cause, and none of these, do I believe, were lost from any fault of the materials used or the mode of applying, but was due to the unfavorable condition of the health or habits of the patient.

Now as to the manner of capping the pulp, If it is freshly exposed or has been restored to a healthy condition, I touch the exposed point with full strength carbolic acid; without wiping this away, I take just enough os-artificial, mixed as thin as I can manage it conveniently, to cover the exposed point thoroughly; I place the cement in the cavity, nearly touching the pulp; then with a small piece of bibulous paper introduced with a spoon shaped excavator, I press the os-artificial gently into accurate contact with the exposed surface of the pulp and the adjacent dentine. The paper at the same time absorbs all the excess of chloride of zinc, supposed to be so deleterious to the pulp by some.

The frightful pain we hear so much about as resulting from the contact of oxy-chloride of zinc with the sensitive pulp is either much diminished by this manner of applying the paste or else I have a remarkably tolerant class of patients, as they generally answer, upon enquiry that the pain is of no consequence. To this thin layer of the cement, I add as much more as may be required, putting in very little at a time, and quickly absorbing the excess of chloride with paper, as I press it firmly into place, and instead of making it as thin as I can handle it, as the first portion applied, I now make it as thick as it can be used. In this way the os-artificial is made so hard that no delay is necessary if it is desirable that the gold filling should be put in at the same sitting. I am thus specific in details, because so many complain of failure and declare that os-artificial, especially, is unfit to be placed in direct contact with an exposed tooth pulp; and from what I have been able to learn from the dental journals, societies and individuals, after a very general abandonment of os-artificial, the entire vegetable, animal and mineral kingdoms have been searched for some thing better, but with no abatement in the cry of failure.

If I am correct in my view of the situation, and find from actual experience, that so soon as the pulp is brought to a condition fit to be sealed up at all, that the plan here described is all that can be desired, I think it is time the wanderers were being called back to something better than they claim to have found in all their search beyond.

The foregoing is applicable after the pulp has been brought to a condition that will admit of capping. To illustrate how I generally proceed with an inflamed or suppurating pulp, I will give my treatment of a case which I consider a most thorough test, so far as one case can be, of its utility.

August, 1875, Miss S. called with pulps exposed in posterior proximal surfaces of the inferior biscuspids of each side. The adjoining surfaces of the molars were in like condition, making four pulps exposed, facing each other in pairs. Three of the cavities extended under the gum; one pulp alone, in a bicuspid was freshly exposed, the other three were supurating or had morbid growths extending outside of the pulp chamber, that bled freely when pierced with an excavator. The pain had become unendurable, hence her visit to the office, This will be recognized as a discouraging case. I told the patient she had delayed treatment too long, and that I did not desire to undertake the case. She begged that I would try to save them, promising perfect obedience to my directions while under treatment.

Being in lower teeth, I, as is my general choice, preferred saving the pulps alive, to devitalizing, and filling roots; that is, I consider it easier to save pulps alive, than to devitalize, remove them, and fill the roots. But this of course should not influence one to adopt a plan of treatment if not best for the patient; here, however, duty and convenience go together. I depleted and cut off the fungus growths that extended out of the pulp chambers, and with a sharp Scranton drill, much larger than the opening to the pulp cavity, I trimmed, smoothed and enlarged this opening, thus relieving pressure upon the swollen pulps and giving more space for the application of the anodynes, etc. In this case I applied dental pain obtunder for a few minutes to allay pain; after this was done, I touched the exposed points with carbolic acid, made a paste of phosphate of lime and lactic acid, and filled each cavity about half full with the paste, in contact with the pulp and sealed up with sandarach varnish and cotton. Dismissed her for three days, if the pain did not require an earlier return. She appeared on the third day, reporting no pain except about one hour the first night. Pulps bled very slightly upon puncturing them a little to ascertain their condition. Applied carbolic acid and sealed up as before with a fresh mixture of lacto-phosphate of lime in contact with the pulps and waited four days. No pain this time; pulps assuming the healthy pink color indicating normal circulation and absence of inflammation. I now capped them with os artificial, in the manner already described in this paper, and one week following, filled them permanently. Have had opportunity to examine these teeth several times since treating and filling. There is no tenderness or sensitiveness to thermal changes distinguishing them from those adjoining that have never been diseased. The impediments to a successful result in this case were the long time the pulps had been exposed, the irritable condition of her nervous system from the protracted pain, the extension of the decay under the gums, and the distance, sixteen miles, to be traveled at each sitting. The advantages were youth and vigorous general health, sanguine temperament, regular habits and absolute obedience to my directions while treating.

The result is no more than a fair illustration of my experience where I have been able to control the patient as to prompt attendance and the avoidance of undue exposure to colds, etc. while treating.

I have always treated these and similar cases presented, as I could get to, not always as I would choose to do if I could control them as would be desirable. For this reason I some times devitalize comparatively healthy pulps with arsenious acid. I confess that only a very short time ago I would have hesitated before making this statement before a state dental association, but not so now. Necessity compelled me to act contrary to the teachings of those whom I am usually content to follow with few or no questions, until experience proves they are mistaken. Thence after hearing arsenic denounced unreservedly and unanimously every year since joining this association, I do not hesitate to speak the first word in its favor that has been uttered to my knowledge in the association since its organization; and although I would, and do preserve every pulp in which the condition and habits of the patient gives the least promise of success. But when I know a patient will not second my efforts, I do not propose to treat his case for weeks or months, to fail at last from his negligence, when arsenic and two sittings will give the result that would be reached at last through no fault of mine; and yet I may suffer the loss of his confidence because of the failure to attain my first purpose.

I go still further, if the pulp is to die at all, it is better that this end is reached through the effects of arsenic than from any other cause known to dental pathology or therapeutics. And why? Because, in a large proportion of cases in all the teeth, but in molars especially, with the best instruments ever invented, you can not always be certain that you remove all the decomposing pulp, and if it has died from inflammation and any considerable portion of it is left in the canals, trouble may be generally expected. If, however, before death, it has been subject to the catalytic action of arsenic, it is very seldom that any trouble will result from any portion that must be left in canals too small or tortuous for removing the devitalized portion remaining therein. The arsenic destroys the morbific agencies in the blood of the thread like remains of the pulp, and conditions or materials for putrefaction, I believe, are as absent as if they had never existed in the canals. It has been the generally accepted theory that arsenic will produce periostitis if not speedily removed after being used to destroy the vitality of the dental pulp. I deny this. It is the closure of the pulp cavity confining the gases that form in this condition of the pulp that produces the irritation resulting in periostitis, not the action of arsenic at all. To prove this: twenty-four or forty-eight hours after the first application of arsenic, open the pulp cavity and allow the escape of the pent up gases, put in more arsenic or not, as you think will be the better test, seal up carefully, as at first; and you will find no trouble or periostitis about that tooth as long as the stopping is intact. Of course, if periostitis has been established even in a moderate degree before the first application of arsenic, that will not stop the inflammation, and some will charge it to the arsenic. Some may feel shocked to hear such statements as this, and will fear the flood gates of empiricism are being lifted from their hinges. I will say, however, that we have had just enough dogmatism in our profession as in all others to frighten off those who doubt certain notions of principles and practice.

As to preserving the vitality of a portion of a tooth pulp, after a considerable portion has been lost from any cause, my experience gives no encouragement that the efforts in that direction can be made so generally successful as to be worth our time and trouble. Three or four years ago much was said and promised of saccharated pepsin for this purpose. After about one year experimenting with it, I did not succeed in digesting away the devitalized portion of a single pulp so far as I could discover. So little has been said about it of late that I suspect others were as badly disappointed as myself. Still if there is any one present who is in the habit of preserving the vitality of a small portion of the pulp, I shall be glad to hear from him.

On the subject of alveolar abscess I shall have very little to say. The last few years have brought very decided advances in treatment of these cases, and so much has been put upon record in reference thereto, that I hope very few have been so unworthy their profession as to remain in ignorance of the successful management of nearly all cases where the co-operation of the patient can be secured during the term of treatment. The old fancy about a "pus secreting sac" I suppose is about abandoned by the studious and thoughtful portion of the profession. A mere reference to this "time honored" myth reminds us how much of our early education must be unlearned as time brings its never ending train of relations.

"True" alveolar abscess, except in cases of extreme scrofulous or syphilitic diathesis, will subside with little or no treatment upon the removal of the cause, and that is a foul vacuum in the roots of the teeth, for which nature seems to have rather more than her traditional horror, but against which it has no means of defense short of the destruction and obliteration of the tooth. Fill this vacuum accurately and innocuously and the abscess will cease to exist for want of an exciting cause. Cure can be hastened by the use of antiseptic, escharotic and stimulant injections.

In filling such roots, I first remove all decomposing matter with a hooked pointed nerve instrument, with drawn temper formed like No. 2 of Dr. Arrington's set. By patient and careful probing I ascertain the length of the canal, and mark this upon the filler to guide me in locating the first installment of filling at the apex of the root. Disinfect with carbolic acid, and with one of the instruments just mentioned, with hook off and the point flattened, and a very slight wrapping of cotton, carry and inject to the apex os-artificial, mixed quite thin, and before it begins to set, I detach the cotton and pack it upon the os-artificial that has gone before; then proceed in the same way to fill the remainder of the canal. Should this alone prove insufficient to stop the discharge, or if I wish to expedite a cure, I make an opening with a lancet and a Scranton drill, about No. 15 of White's burr gauge, to the point of the root, and insert a tent of cotton charged with carbolic acid. With this plan of treatment, I am so nearly uniformily successful, that I feel no need of a better way.

In the treatment of what has been denominated "false alveolar abscess," that which forms below the apex of the roots, and discharges at the necks of the teeth, for the recession of the gums produced by lime deposit, I also have a favorite, or what I consider a best way of proceeding. It is to remove with instruments, the deposit upon the necks and roots of the teeth as thoroughly as possible; with a pleget of cotton and a suitable instrument I carry to every effected part, aromatic sulphuric acid. A very few repetitions of this remedy from three days to a week apart will generally clear the way for nature to repair the damage, except in aged patients or those of anæmic habits, or other constitutional weakness, and then we can have those much coveted consultations with the general practitioner of medicine. Careful, gentle and thorough brushing of the lower gums upward and of the upper ones downward, must be earnestly enjoined.

For antiseptic, narcotic, stimulant and escharoctic purposes it will be noticed that I have given preference to carbolic acid over the many other agents in common use for these purposes. Between it and creosote, I have never been able to note a clearly defined difference in results that some claim to have observed. Glycerole of thymol and salicylic acid I have not found so generally applicable or reliable, but use them occasionally interchangeably with carbolic acid where a number of applications are required.

It is very seldom I suppose that dentists are consulted in the most serious pathological condition of the human teeth that is experienced so generally in first dentition. Notwithstanding this fact, I hope to do some good in calling your attention to, in my mind, a most important practice in treating this often fatal disease of childhood. You may use it in your own families if not called to treat, or have no means of extending the treatment to others. It is lancing the gum over the erupting teeth in the order in which they should make their appearance. My observation is that physicians are very lax or sparing in the use of the lancet in treating these cases. They are so unfamiliar with the order of the eruption of the teeth, that often times the child is in the agony of death before the tumefaction of the surface shows them where to operate; and there has been so much said against the practice, so much about the danger from hæmorrhage, that when a simple incision in the right spot would save a life, they neglect the only possible remedy. I speak positively. Watch your child until the palor of death has begun to settle upon it, under exclusive drug treatment, then see it quickly relieved and reviving by the use of the lancet, and you will be positive in your feelings and expressions on the subject ever afterward. Lancing early and often as a good dentist would know when, where and how to do it is as near a specific as can be found in the entire field of therapeutics. It is based upon the principle of removing the cause, a principle you will notice I have endeavored to keep in view in each division of this paper. After thus removing the cause of the disturbance much more may be done that would be well nigh useless if not preceded by the lancet.

Like other points touched upon in this paper, this very brief reference to lancing the gums of teething children must suffice. Those desiring to study the matter further, I would refer to Prof. Flagg's papers in the *Dental Cosmos*, as among the most worthy of their attention. Then as the next step I would suggest that the regulars be stirred up to a sense of their delinquency in a practice that would save many precious lives they now allow to be lost.

